# Capturing Dynamic Performance in a Cognitive Model: Estimating ACT‐R Memory Parameters With the Linear Ballistic Accumulator

**DOI:** 10.1111/tops.12614

**Published:** 2022-05-09

**Authors:** Maarten van der Velde, Florian Sense, Jelmer P. Borst, Leendert van Maanen, Hedderik van Rijn

**Affiliations:** ^1^ Department of Experimental Psychology, Behavioural and Cognitive Neuroscience University of Groningen; ^2^ Bernoulli Institute, Department of Artificial Intelligence University of Groningen; ^3^ Department of Experimental Psychology Utrecht University

**Keywords:** Memory, Dynamic performance, Individual differences, Cognitive modeling, ACT‐R, Linear ballistic accumulator

## Abstract

The parameters governing our behavior are in constant flux. Accurately capturing these dynamics in cognitive models poses a challenge to modelers. Here, we demonstrate a mapping of ACT‐R's declarative memory onto the linear ballistic accumulator (LBA), a mathematical model describing a competition between evidence accumulation processes. We show that this mapping provides a method for inferring individual ACT‐R parameters without requiring the modeler to build and fit an entire ACT‐R model. Existing parameter estimation methods for the LBA can be used, instead of the computationally expensive parameter sweeps that are traditionally done. We conduct a parameter recovery study to confirm that the LBA can recover ACT‐R parameters from simulated data. Then, as a proof of concept, we use the LBA to estimate ACT‐R parameters from an empirical dataset. The resulting parameter estimates provide a cognitively meaningful explanation for observed differences in behavior over time and between individuals. In addition, we find that the mapping between ACT‐R and LBA lends a more concrete interpretation to ACT‐R's latency factor parameter, namely as a measure of response caution. This work contributes to a growing movement towards integrating formal modeling approaches in cognitive science.

## Abstract 2

The parameters governing our behavior are in constant flux, and capturing these dynamics in cognitive models remains a challenge. We demonstrate how a mapping between ACT‐R's model of declarative memory and the linear ballistic accumulator enables efficient estimation of memory parameters from data. The resulting estimates provide a cognitively meaningful explanation for observed differences in behavior over time and between individuals.

## 1 Introduction

Cognitive architectures such as ACT‐R (Anderson, 2007) provide a framework for developing models of cognition. A challenge commonly faced by modelers is to accurately capture changes in cognitive performance over time, as well as individual differences between people, in the parameters of such models. Current approaches tend to rely on computationally expensive and statistically suboptimal methods like parameter sweeps to identify the best‐fitting parameter values. Mathematical modeling methods can serve as a more efficient and rigorous alternative (Fisher, Houpt, & Gunzelmann, 2020). In this paper, we contribute to previous efforts to connect cognitive architectures and mathematical modeling by using the linear ballistic accumulator (Brown & Heathcote, 2008) to infer ACT‐R parameters governing memory retrieval.

Traditionally, research in cognitive modeling has taken many different forms, often with relatively little overlap in methodology and terminology, making it challenging to integrate and compare approaches (Gluck, Bello, & Busemeyer, 2008). Two of the dominant paradigms are the cognitive architecture approach and the mathematical modeling approach. On the one hand, there is a rich history of model development in the context of cognitive architectures like ACT‐R (Anderson, 2007), which provide a general‐purpose framework suitable for constructing detailed, integrative models of complex behavior, ranging from multitasking and skill acquisition to reasoning and mind wandering (Gluck & Pew, 2005; Salvucci & Taatgen, 2008; van Vugt, van der Velde, & ESM‐MERGE Investigators, 2018). On the other hand, mathematical models of cognition—such as the family of evidence accumulation models, which include the linear ballistic accumulator (Brown & Heathcote, 2008) and the drift diffusion model (Ratcliff, 1978)—have proven invaluable in modeling performance on a wide array of cognitive tasks in a statistically rigorous and scalable way (Heathcote et al., 2019). While work in both paradigms has mostly happened in parallel, there have been attempts at integrating the two,  for example, Fisher et al. (2020), Nicenboim and Vasishth (2018), and van Maanen, van Rijn, and Taatgen (2012). As Fisher et al. (2020) point out, such integration of approaches can lead to new insights and can help promote a common language across modeling traditions. Here, we explore how a convergence of the ACT‐R cognitive architecture and the linear ballistic accumulator helps in modeling dynamic performance in the domain of memory retrieval.

### 1.1 Memory retrieval as evidence accumulation

Retrieval of information from memory can be viewed as a process of evidence accumulation, in which internal and external cues contribute evidence to candidates in memory that are competing for retrieval (Anderson, 2007; Ratcliff, 1978). The first candidate to accumulate enough evidence to cross a boundary wins the race and is retrieved. The dynamics of this process are determined by the amount of evidence each candidate needs to accumulate to cross the boundary and the rate at which this evidence accumulates.

While evidence accumulation models have seen most use in the domain of decision‐making (e.g., Brown & Heathcote, 2008; Ratcliff, Smith, Brown, & McKoon, 2016; Smith & Ratcliff, 2004; Usher & McClelland, 2001), there have been some applications in the domain of memory retrieval. Van Maanen et al. showed that a leaky competing accumulator model could explain performance in picture‐word interference tasks (van Maanen & van Rijn, 2007; van Maanen et al., 2012). In this model, memory chunks accumulate activation by receiving positive and negative spreading activation from other chunks. More recently, Nicenboim and Vasishth (2018) and Fisher et al. (2020) implemented the ACT‐R model of declarative memory in a lognormal race model (LNR; Rouder, Province, Morey, Gomez, & Heathcote, 2015), in which the rate at which evidence for a chunk accumulates depends on its activation.

Here, we extend this formalization of ACT‐R memory retrieval as an LNR to a more flexible linear ballistic accumulator model (LBA; Brown & Heathcote, 2008). Unlike the LNR, the LBA is able to estimate the rate of accumulation separately from the distance accumulators need to travel to reach the decision boundary. As we will discuss more extensively later, this is useful, because both accumulation rate and distance to the boundary have natural counterparts in ACT‐R: the accumulation rate corresponds to the activation of the chunk, while the distance can be linked to the latency factor (*F*) parameter. As such, the LBA provides a cognitively meaningful interpretation of ACT‐R's *F* parameter as an inverse measure of response caution—the larger the distance, the more evidence needs to be collected before a response is made—and offers a method by which it can be estimated from response data.

In the following sections, we first describe the formal link between ACT‐R's declarative memory and the LBA. We then demonstrate how the LBA can be used to recover ACT‐R parameters in a simulation study. Finally, we fit the LBA to an empirical dataset, showing how it can offer insight into the mechanisms underlying changes in retrieval performance over time.

## 2 Casting ACT‐R's declarative memory as a linear ballistic accumulator

The linear ballistic accumulator model (Brown & Heathcote, 2008) proposes that response behavior can be explained through a race between accumulators. Each accumulator has a certain amount of starting evidence *k* that increments linearly at a drift rate *v* until it reaches a decision boundary *d*. The first accumulator to reach the boundary determines the response choice and latency. A constant non‐decision time *t*_0_ is also added, representing the time required for other components of the response process, such as perceptual and motor functions. There are two sources of variability between trials: the starting point *k*, which is typically drawn from a uniform distribution [0, *A*], and the drift rate *v* for each response option *i*, which is often drawn from a normal distribution $N(v_{i},s_{i})$. The LBA assumes a constant rate of evidence accumulation over a trial, so the time required for an accumulator *i* to reach its boundary on a trial *j* is the distance $d-k_{j}$ divided by the drift rate, plus non‐decision time:

(1) $$RT_{ij}=\frac{d-k_{j}}{v_{ij}}+t_{0}.$$

Across trials, the average starting point is A/2 and the average drift rate for an accumulator *i* is $v_{i}$, so the expected finishing time is

(2) $$E\left(RT_{i}\right)=\frac{d-A/2}{v_{i}}+t_{0}.$$

We can map the LBA parameters onto ACT‐R memory parameters. ACT‐R models declarative memory as a set of symbolic chunks, each with a subsymbolic activation that decays over time and is subject to noise (Anderson, 2007). The time required to retrieve a chunk depends on its activation: the more active the chunk, the faster its retrieval will be. Just as in the LBA, the time course of memory retrieval is deterministic once the starting values are known. If multiple chunks match a retrieval request, the chunk with the highest activation at the time of the request—and therefore the lowest retrieval time—wins. A full response also involves non‐memory operations, such as stimulus encoding and response execution, which can be captured by adding a term $t_{er}$ to the retrieval time. This term fulfills the same role as the *t*_0_ parameter in the LBA.

ACT‐R defines the full time required to retrieve a chunk *i* with an activation *A* and respond accordingly by the following equation, in which *F* is the latency factor, a positive scaling parameter^1^:

(3) $$RT_{i}=F*e^{-A_{i}}+t_{er}.$$

By default, ACT‐R represents *F* as a constant. However, to match the structure of the LBA, we assume here that *F* is a variable between trials. Similar to the starting point *k* in the LBA, *F* is drawn from a uniform distribution [$a_{F},b_{F}$] in every trial. In addition, following Lebière, Anderson, and Reder (1994), we assume normally distributed trial‐to‐trial variability, or noise, in the activation of chunks: $A_{i}∼N\left(\mu_{i},\sigma_{i}\right)$. The time required to retrieve chunk *i* on trial *j* and respond accordingly therefore becomes

(4) $$RT_{ij}=F_{j}*e^{-A_{ij}}+t_{er}.$$

Across trials, the average latency factor is $\bar{F}=\frac{a_{F}+b_{F}}{2}$ and the average activation is $\mu_{i}$, so the expected finishing time is

(5) $$E\left(RT_{i}\right)=\bar{F}*e^{-\mu_{i}}+t_{er}.$$

We can rewrite this equation in a similar form to (2):

(6) $$E\left(RT_{i}\right)=\frac{\bar{F}}{e^{\mu_{i}}}+t_{er}.$$

The mapping between ACT‐R's parameters (left) and those of the LBA (right) then becomes straightforward:

(7) $$\begin{array}{cc} \bar{F} & =d-A/2, \end{array}$$

(8) $$\begin{array}{cc} \mu_{i} & =\ln(v_{i}), \end{array}$$

(9) $$\begin{array}{cc} t_{er} & =t_{0}. \end{array}$$

With this mapping, ACT‐R's mean latency factor ($\bar{F}$) corresponds to the average distance between the starting point and boundary in the accumulator model, often conceptualized as the response to caution. This means that, given a constant activation, a higher value of $\bar{F}$ means that more evidence is required to complete a retrieval. Fluctuations in this parameter between trials can be thought of as representing moment‐to‐moment changes in response caution (Boehm, van Maanen, Forstmann, & van Rijn, 2014). By default, ACT‐R assumes a fixed *F* parameter, which means that all variability in retrieval time over the duration of a task is ascribed to activation and activation noise. This assumption can lead to incorrect conclusions in scenarios where we cannot assume consistent response caution between trials, for example, due to post‐error slowing (Dutilh et al., 2012) or shifts in the speed‐accuracy trade‐off (van Maanen et al., 2011). By assuming a variable, rather than a fixed, *F* parameter, we can distinguish such fluctuations in response caution from general activation‐related variability.

The mapping relates the mean activation $\mu_{i}$ of a chunk to the logarithm of the mean drift rate $v_{i}$, meaning that a highly activated chunk can be seen as accumulating evidence more rapidly than one with a lower activation. Put differently, the drift rate $v_{i}$ is equivalent to $e^{\mu_{i}}$, the odds of needing the chunk.

Finally, there is a direct equivalency between the non‐retrieval time ($t_{er}$ and *t*_0_) in both models.

**Fig. 1:**
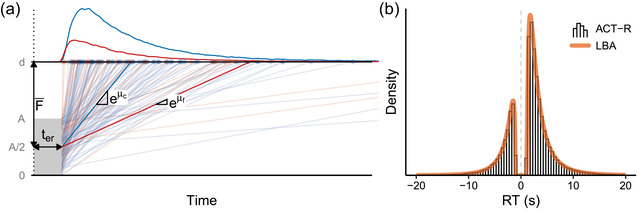
Casting ACT‐R memory retrieval as a linear ballistic accumulator. (a) ACT‐R retrieval with two competing chunks visualized as an LBA, with marginal RT distributions shown at the top; see the main text for details. (b) RT distributions of an ACT‐R model (histogram) and the equivalent LBA model (orange curve). Error responses are shown as negative RTs.

Fig. 1a visualizes the ACT‐R retrieval process in the style of an accumulator model. It shows two chunks, *c* (blue) and *f* (red), competing for retrieval over multiple trials. In each trial, both accumulators race to cover the vertical distance to the boundary. The winner gets retrieved in the time it takes to reach the boundary. The vertical distance is shared among the accumulators but varies between trials, following a uniform distribution. Furthermore, there is normally distributed trial‐to‐trial variability in the activation of the chunks, and therefore in the rate at which each chunk accumulates evidence. Given that activation has a logarithmic relation to the drift rate in the proposed mapping, drift rates now follow a lognormal distribution (see Terry et al., 2015, for more detail on non‐normal drift rate distributions). Fig. 1b demonstrates that ACT‐R and the LBA generate identical response time distributions for a given set of parameters when using the mapping in (7)–(9). Interactive versions of these figures, in which the model parameters can be freely adjusted, are available at https://cogmod.shinyapps.io/actr‐lba/.

## 3 Simulation: Recovering ACT‐R parameters

Given this mapping, it should be possible to identify ACT‐R memory parameters from response data (RT and choice) using existing methods for fitting the LBA. Therefore, we performed a simulation study with two goals: to investigate whether the LBA can recover ACT‐R memory parameters from a typical participant sample completing a reasonable number of trials and to ensure that parameter recovery works regardless of specific parameter values. The code required to reproduce this simulation study is available at https://osf.io/wpvj7/.

### 3.1 Data

ACT‐R was used to simulate 25 distinct model participants, each performing a sequence of retrieval trials. Retrieval was modeled as a competition between two chunks, *c* and *f*, representing a correct and incorrect response to a retrieval cue, respectively^2^. In each trial, the response time was the time (in seconds) required for the winning accumulator to reach the decision boundary. For each model participant, ACT‐R parameters were sampled randomly from plausible distributions, listed in Table 1. To ensure that parameters recovered by the LBA would all be on the same scale as the original ACT‐R parameters, we fixed the standard deviation of the activation of the correct response ($\sigma_{c}$) to 1, both in ACT‐R and in the LBA^3^.

**Table 1:** ACT‐R parameters used in the simulation study

	Description	Distribution
$\mu_{c}$	Mean activation of correct answer	$\mu_{c}∼N_{-}\left(-.5,.5\right)$
$\mu_{f}$	Mean activation of incorrect answer	$\mu_{f}∼N_{-}\left(-1.5,.5\right)$
$\sigma_{c}$	SD of activation of correct answer	$\sigma_{c}=1$
$\sigma_{f}$	SD of activation of incorrect answer	$\sigma_{f}∼N_{+}\left(1.5,.5\right)$
$\bar{F}$	Mean latency factor	$\bar{F}∼N_{+}\left(1,.5\right)$
$R_{F}$	Range of latency factor ($b_{F}-a_{F}$)	$R_{F}∼N_{+}\left(.1,.05\right)$
$t_{er}$	Non‐retrieval time	$t_{er}∼N_{+}\left(.75,.5\right)$

*Note*. $N_{+}$ and $N_{-}$ are truncated normal distributions, limited to positive and negative values, respectively.

We repeated the process with differently sized datasets, ranging from 25 to 50,000 trials per participant, to gauge the effect of dataset size on recovery accuracy.

### 3.2 Model fitting

The LBA was fitted separately to each model participant's responses using the *nlminb* optimizer in R (version 3.6.3; R Core Team, 2020). We ran this optimizer 250 times with randomly generated starting values and kept only the parameter values that yielded the highest summed log‐likelihood across all runs. The *dLBA* density function from the *rtdists* package (version 0.11‐2; Singmann, Brown, Gretton, & Heathcote, 2020) served as the objective function. For each model participant, we derived individual ACT‐R parameters from the best‐fitting LBA using the mapping in (7)–(9).

### 3.3 Results

**Fig. 2:**
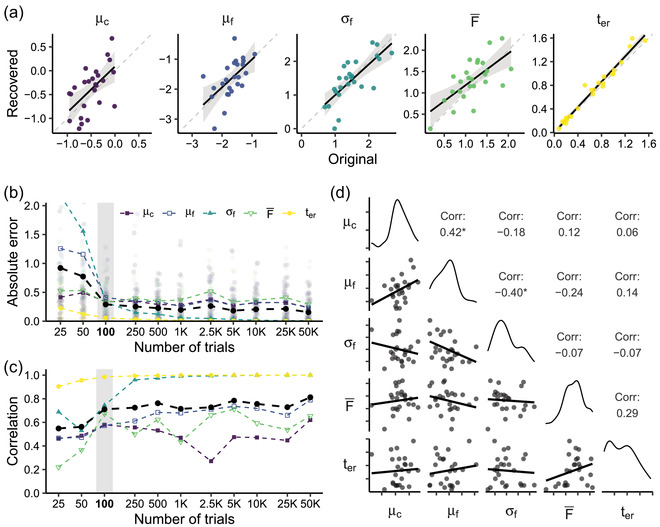
Recovery of ACT‐R parameters using the LBA. (a) Original versus recovered parameter values for a dataset with 100 trials per participant. Parameter descriptions are given in Table 1. (b) Recovery accuracy (absolute error) for datasets with different numbers of trials per participant. Light‐colored points show individual errors, dark‐colored points show the mean error per parameter, and black points show the mean error across parameters. The gray bar marks the dataset shown in subfigure a. (c) Correlation (Pearson's *r*) between original and recovered parameter values for datasets with different numbers of trials per participant. Colored points show the correlation per parameter, and black points show the mean correlation across parameters. The gray bar marks the dataset shown in subfigure a. (d) Correlations (Pearson's *r*) between pairs of recovered parameters in the dataset with 50,000 trials per participant.

The results of the parameter recovery process are shown in Fig. 2. As Fig. 2a indicates, original parameter values could already be recovered with reasonable accuracy from a dataset with 100 trials per participant.

Figs. 2b and 2c show how recovery accuracy changed as a function of dataset size, both in terms of the absolute error of recovered parameter values relative to the original values (Fig. 2b) and in terms of the correlation between original and recovered values (Fig. 2c). Unsurprisingly, recovery accuracy improved when there were more trials constraining the fit, though the current fitting method reached a plateau once there were at least 250 trials per participant.

Given a sufficient number of observations, $\sigma_{f}$ and $t_{er}$ could be recovered with very high accuracy. The mean latency factor $\bar{F}$ and the activation means $\mu_{c}$ and $\mu_{f}$ remained more difficult to recover, suggesting a degree of trade‐off between parameters: variability in one parameter could to some extent also be captured by adjustments to another parameter (see also, e.g., Miletić, Turner, Forstmann, & van Maanen, 2017). Fig. 2d confirms that there were indeed correlations between some of the recovered parameters in the largest dataset we tested, which contained 50,000 trials per participant: the mean activation estimates of the two chunks were positively correlated, and the mean activation of the incorrect chunk correlated negatively with the activation noise of that chunk.

## 4 Example application: Modeling changing retrieval performance in empirical data

To demonstrate how the method may be used to explain dynamic memory performance in terms of cognitively meaningful constructs, we fitted the LBA to empirical data from a multisession retrieval practice task.

### 4.1 Data

We used data from a retrieval practice task completed by recruits of the Commando Corps, Royal Netherlands Army (*Korps Commandotroepen*), in which participants learned the names of fictitious safe houses on a map (van der Velde et al., 2020, 2021). The data were collected as part of a larger project; see Huijzer et al. (2022) for a more detailed description of the participants and procedure. The task consisted of a sequence of retrieval practice trials. In the first presentation, a safe house was shown with its name, while subsequent repetitions required participants to select the correct name themselves from a set of four answer options. Participants completed three 8‐min sessions over the course of a week. They studied a different map in every session, and maps were counterbalanced between participants. The task was presented within an adaptive learning system that schedules each item to be repeated whenever its activation is expected to hit a threshold (Sense, Behrens, Meijer, & van Rijn, 2016; van der Velde, Sense, Borst, & van Rijn, 2021). As such, we could expect the activation of the chunks being retrieved to be fairly stable across trials, despite the novelty of the material. Response accuracy and response time were recorded in every trial.

Session 1 was scheduled on the first day of the week, while the second and third sessions took place several days later and were scheduled immediately before and after a high‐intensity loaded speed march of about 40 min. We expected performance to change for two reasons: increased familiarity with the task might lead to better performance after the first session, and the physical exertion of the speed march might affect performance in the third session. As such, the dataset provides an excellent test case for our method.

For the analysis, we removed the first trial for each item (in which the answer was shown on screen), trials in which participants did not respond within 30 s, and trials in which the recorded response time was lower than 300 ms. Since the simulation study showed that recovery was worse in small datasets, participants had to have completed at least 50 practice trials per session to be included. In addition, we required that participants made at least five error responses per session, to give the model a chance of fitting the error response distribution. These criteria struck a balance between ensuring a sufficient number of observations per participant and including as many participants as possible. They yielded a dataset with 12,568 usable observations (out of 29,441) from 50 (out of 127) participants.

### 4.2 Model fitting

We fitted the LBA separately to each of the three retrieval practice sessions for each participant. The fitting procedure was the same as in the simulation study. The analysis code is available at https://osf.io/wpvj7/.

### 4.3 Results

**Fig. 3:**
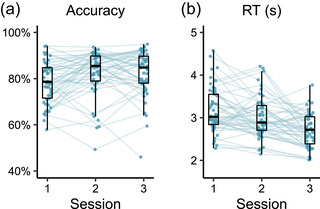
Performance on the retrieval practice task by participant. (a) Percentage correct responses per session. (b) Median response time on correct responses per session.

Fig. 3 shows participants' performance on the task over the three sessions. Despite the task difficulty being the same in all three sessions, performance improved in two ways. First, response accuracy increased and then plateaued: a logistic mixed‐effects model with a main effect of session^4^ and random intercepts for participants showed that accuracy increased from the first to the second session (z=−4.680, p<.001), but found no evidence for a change from the second to the third session (z=−0.253, p=.8). Second, responses became faster: a generalized mixed‐effects model with a Gamma link function and with a main effect of session and random intercepts for participants found a decrease in response times on correct trials from Session 1 to Session 2 (t=2.250, p=.0244) and from Session 2 to Session 3 (t=−7.182, p<.001).

**Fig. 4:**
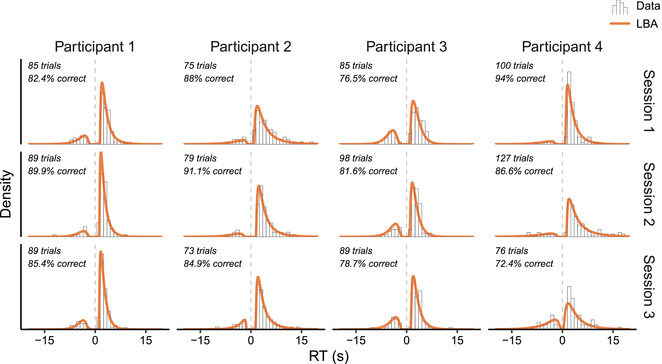
Best fits of the LBA to the response data of four participants over three retrieval practice sessions. Error responses are shown as negative RTs. The number of available trials and the response accuracy is shown in the top left corner of each plot.

Fig. 4 shows the best fit of the LBA to the response time distributions of four randomly selected participants. These examples suggest that the model captured the shape of the data quite well, although the low number of trials and high response accuracy did make it challenging to fit the error responses.

**Fig. 5:**
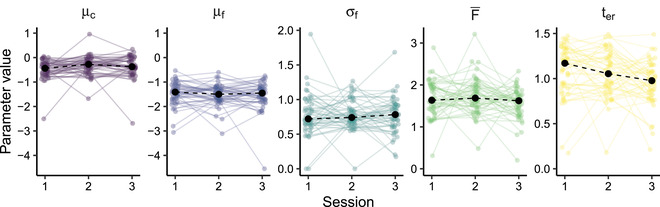
ACT‐R memory parameters inferred from the data. Colored points show individual estimates; large black points indicate the median value across participants. Parameter descriptions are given in Table 1.

The inferred ACT‐R parameters for all participants are shown in Fig. 5. There is substantial variation in the parameter values for individual participants, but they are nonetheless clustered quite neatly around the sample averages. As one would expect, the activation of the correct answer ($\mu_{c}$) tended to exceed the activation of the incorrect answer ($\mu_{f}$), reflecting participants' better‐than‐chance performance. The estimates of the mean latency factor ($\bar{F}$) are similar to values identified by others (e.g., see Table 4 in Anderson, Bothell, Lebiere, & Matessa, 1998). We found a non‐retrieval time ($t_{er}$) of about 1 s, which we consider a plausible estimate of the time needed to encode a complex visual stimulus and execute a response.

To explore possible changes in parameter values over time, we fitted separate linear mixed‐effects models to each parameter, testing whether there was a session effect on the parameter value, with random intercepts for participants. These models suggested that the parameters generally remained fairly constant between sessions^5^. However, the activation of the correct answer ($\mu_{c}$) did appear to increase from Session 1 to Session 2 (t(98)=−2.050, p=.043). Furthermore, since the outcome of the retrieval depends on which of the two candidate chunks has the highest activation, rather than on the individual activation of either chunk, we also fitted a linear mixed‐effects model with the difference in activation $\mu_{c}-\mu_{f}$ as the dependent variable. This model similarly suggested that the activation difference was higher in Session 2 than in Session 1 (t(98)=−3.133, p=.00228), indicating that, on average, participants' chances of retrieving the correct answer improved. Finally, the non‐retrieval time $t_{er}$ showed a significant decrease from Session 2 to Session 3 in particular (t(98)=−2.351, p=.0207), reflecting a speed‐up in perceptual and/or motor functions. Others have reported a similar reduction in this parameter as a result of practice (e.g., Dutilh, Vandekerckhove, Tuerlinckx, & Wagenmakers, 2009).

In conclusion, exploratory analysis of the inferred ACT‐R parameter estimates suggests that the observed increase in accuracy and response speed from Session 1 to Session 2 is the result of a higher mean activation of the correct answer and a greater difference in activation between the correct and incorrect answer, while the drop in response times from Session 2 to Session 3 may be attributable to a decrease in non‐retrieval time $t_{er}$.

## 5 Discussion

We have demonstrated a mapping of the parameters of the linear ballistic accumulator onto parameters governing declarative memory retrieval in ACT‐R. By fitting the LBA to retrieval data and mapping the inferred LBA parameters onto ACT‐R memory parameters, we can arrive at a mechanistic explanation for observed performance changes, without needing to build and fit an ACT‐R model directly. The resulting ACT‐R parameters—activation of chunks, duration of non‐retrieval processes, and latency factor—have cognitively meaningful interpretations within the wider context of the architecture, enhancing the interpretation that could be given by the LBA alone. The mapping extends upon an earlier mapping between the lognormal race model and ACT‐R (Nicenboim & Vasishth, 2018), by adding the ability to fit the latency factor.

From a theoretical standpoint, ACT‐R benefits from this connection to the LBA too: the latency factor is given a more concrete meaning, namely as a (variable) measure of response caution. The mapping requires that this latency factor is variable between retrieval attempts instead of fixed, as it would be by default in ACT‐R. As Fisher et al. (2020) suggest, adapting the assumptions made by ACT‐R can be appropriate if doing so facilitates the integration of modeling approaches. Furthermore, we argue that this change makes for a better model of retrieval, as it enables ACT‐R to separate between‐trial variability in memory activation from variability in response caution. This makes it possible to capture trial‐to‐trial shifts in participants' speed‐accuracy trade‐off, which is a key advantage of evidence accumulation models that has been difficult to replicate in ACT‐R (Brown & Heathcote, 2008). Previously, Schneider and Anderson (2012) modeled the speed‐accuracy trade‐off in ACT‐R by assuming an additional deadline‐induced guessing process, but such a mixture of processes appears to lack empirical support (van Maanen, 2016). Modeling speed‐accuracy trade‐off through a variable *F* parameter may therefore be a good alternative.

An important limitation of our method is that it assumes that the distribution of drift rates—and therefore the activation of memory chunks—remains constant across a block of trials. This assumption is most likely to be met when information is so ingrained that there is no appreciable decay in its activation, for example, in sentence processing (Nicenboim & Vasishth, 2018), or when retrieval attempts are timed such that they occur whenever a particular activation is reached (e.g., with adaptive scheduling, as used in our empirical example).

The method described here allows one to disentangle several factors contributing to memory retrieval performance. In many settings, inside and outside the laboratory, the parameters governing our behavior are inevitably in flux: we learn and forget, we become tired or impatient, our goals and desires change, and we let our minds wander. There is clear explanatory power in being able to capture such changes within a mathematical model. Linking the terms of that mathematical model to constructs defined in a cognitive architecture can further aid the interpretation of observed behavior.

The mapping between ACT‐R's declarative memory and the linear ballistic accumulator represents a specific instance in which two different modeling paradigms converge. Such convergences provide an opportunity for modelers from different traditions to express their ideas in a common language and to benefit from the possibilities offered by a different paradigm to their own. For instance, in contrast to ACT‐R, mathematical models like the LBA are well‐suited for Bayesian and hierarchical methods of parameter estimation (Heathcote et al., 2019). We currently used a relatively simple procedure for fitting the LBA, but extending this approach to a hierarchical Bayesian LBA may be beneficial (e.g., Nicenboim, 2018). It would enable modeling multiple participants and sessions simultaneously, improving the ability to estimate and compare participant‐level and group‐level effects, while also capturing the uncertainty in those estimates (Fisher et al., 2020). This could be particularly valuable in datasets with fewer observations per participant, where our current approach still struggles.

In summary, we have demonstrated how a mapping between the ACT‐R cognitive architecture and the linear ballistic accumulator can aid in capturing dynamic performance in a cognitive model, thereby contributing to growing efforts to integrate formal modeling approaches.

## Notes

1 An additional parameter *f* may be used to scale the exponent: $RT_{i}=F*e^{-f*A_{i}}+t_{er}$. This parameter is typically held constant at 1, and we make the same simplification here as it has no bearing on the outcomes.

2 The model presented here does not explicitly account for possible retrieval failures; these are currently lumped together with incorrect retrievals in a single accumulator. Provided that the data being modeled allow for a distinction between these different types of error, an additional accumulator could be added, representing ACT‐R's retrieval threshold τ (Lebière et al., 1994).

3 See Brown and Heathcote (2008) for alternative solutions to the scaling problem in accumulator models. Note that the choice of scaling constraint affects the interpretation of the model parameters (van Maanen & Miletić, 2021).

4 Session 2 was used as the reference level (intercept). Coefficients and corresponding test statistics for Sessions 1 and 3 were estimated relative to this intercept.

5 Aside from the reported effects, there was some evidence for a decrease in the $\bar{F}$ parameter from Session 2 to Session 3, though the corresponding model failed to converge. More generally, these results should be interpreted with a degree of caution, as repeated LBA fits yield slightly different parameter estimates due to random variation in starting values.
